# Brain-derived neurotrophic factor and its receptor in the human and the sand rat intervertebral disc

**DOI:** 10.1186/ar2456

**Published:** 2008-07-17

**Authors:** Helen E Gruber, Jane A Ingram, Gretchen Hoelscher, Natalia Zinchenko, H James Norton, Edward N Hanley

**Affiliations:** 1 Department of Orthopaedic Surgery, Carolinas Medical Center, PO Box 32861, Charlotte, NC 28232, USA; 2 Department of Biostatistics, Carolinas Medical Center, PO Box 32861, Charlotte, NC 28232, USA

## Abstract

**Introduction:**

Brain-derived neurotrophic factor (BDNF) was first identified in the intervertebral disc (IVD) when its molecular upregulation was observed in sections of nucleus pulposus cultured under conditions of increased osmolarity. BDNF is now known to be involved in a number of biologic functions, including regulation of differentiation/survival of sensory neurons, regulation of nociceptive function and central pain modulation, and modulation of inflammatory pain hypersensitivity. In addition, more recent investigations show that BDNF can induce the recruitment of endothelial cells and the formation of vascular structures. The objectives of the present study were to use immunocytochemistry to determine the distribution of BDNF and its receptor (BDNF-tropomyosine receptor kinase B) in the human IVD, and to test for gene expression of BDNF and its receptor in cultured human annulus fibrosus cells.

**Methods:**

We studied immunohistochemical localization of BDNF and its receptor in the human annulus, quantified the percentage of outer annulus and inner annulus cells and nucleus cells positive for BDNF immunolocalization, and studied the gene expression of BDNF and its receptor using microarray analysis.

**Results:**

The percentage (mean ± standard error of the mean) of cells positive for BDNF localization was significantly greater in the outer annulus (32.3 ± 2.7%, n = 22) compared with either the inner annulus (8.1 ± 1.5%, n = 6) or the nucleus (10.4 ± 2.8%, n = 3) (*P *< 0.0001). BDNF-receptor immunolocalization showed a pattern similar to that of BDNF, but was not quantitatively assessed. BDNF gene expression levels from cultured annulus cells showed a significant positive correlation with increasing levels of IVD degeneration (*P *= 0.011).

**Conclusion:**

These findings provide data on the presence of BDNF and its receptor in the human IVD at the translational level, and on the expression of BDNF and its receptor by cultured human annulus cells. Our findings point to the need for further studies to define the role of BDNF in the human IVD and to investigate regulatory events within the disc that control the expression of BDNF and its receptor.

## Introduction

Brain-derived neurotrophic factor (BDNF) was first identified in the intervertebral disc (IVD) when its molecular upregulation was observed in sections of nucleus pulposus cultured under conditions of increased osmolarity by Boyd and colleagues [1]. BDNF is now known to be involved in a number of biologic functions, including regulation of differentiation/survival of sensory neurons, regulation of nociceptive function and central pain modulation, and modulation of inflammatory pain hypersensitivity.

Nerves grow into tissues in response to signaling molecules called neurotrophins, which are involved in the survival, differentiation, migration and outgrowth of neurons. We now know that neurotrophins are expressed in non-neuronal tissues, including the IVD [2-4]. Gigante and colleagues recently reported the presence of nerve growth factor (NGF) mRNA, the high-affinity tyrosine kinase A receptor and the low-affinity p75 receptor in rounded cells in the disc annulus and nucleus pulposus [5]. NGF and its receptors tyrosine kinase A and p75 are expressed not only in the developing nervous system, but also in the mature adult nervous system – where they are particularly important because of their association of primary nociceptive neurons. The recent review by Mendell and colleagues points out that in adults NGF acts as an important intermediate in pain, contributing to peripheral and central sensitization [6].

One other neurotrophin has previously been studied in the IVD. Abe and colleagues have reported on the expression of NGF by human IVD cells in control disc tissue *in vivo *and *in vitro *in cells from control IVDs using immunocytochemistry [7]. NGF was found to be high in the outer annulus and in herniated disc tissue. Abe and colleagues' work also demonstrated that the proinflammatory cytokines IL-1 and TNFα had stimulatory effects on NGF. These authors suggested that such actions may play a role in nerve sprouting into the IVD, and may be associated with discogenic pain.

Ohtori and colleagues have reported on the presence of BDNF in dorsal root ganglion neurons innervating lumbar discs in the rat [8]. Supraspinal BDNF-tyrosine kinase receptor B signaling is now known to represent a mechanism underlying the development of persistent pain [9,10].

Another important role for BDNF involves more recent advances in our understanding of BDNF function. The interesting studies by Kermani and colleagues have documented the ability of BDNF to promote revascularization via endothelial cells and hematopoietic progenitors [11]. These investigators suggest their work with skeletal muscle and heart indicates that the signaling pathway involving BDNF and its receptor may also be active in modulation of angiogenesis in specific organs in the adult. In light of the avascular status of the adult IVD, this role merits future research.

Because of the significance of BDNF in the IVD, our objectives in the present work were to use immunocytochemistry to determine the distribution of BDNF and its receptor in the human IVD, and to test for gene expression of BDNF and its receptor in cultured human annulus fibrosus cells.

## Materials and methods

### Clinical study population

The experimental study of human IVD specimens was approved prospectively by the authors' Human Subjects Institutional Review Board (protocol number 08-04-09E). The need for informed consent was waived (since surgical IVD tissue is routinely discarded at our institution). Surgeries for disc degeneration are routine clinical practice in our hospital. The Thompson grading system is used to score IVD degeneration over the spectrum of stages from Thompson grade I (a healthy disc) to discs with advanced degeneration (Thompson grade V) [12].

Patient specimens were derived from surgical disc procedures performed on individuals with herniated discs and with degenerative disc disease. Surgical specimens were transported to the laboratory (<30 min after surgical removal) in sterile tissue culture medium and were placed in 10% neutral buffered formalin for no longer than 24 hours. Care was taken to remove all granulation tissue and to sample only IVD tissue. Donor disc specimens were obtained via the National Cancer Institute Cooperative Human Tissue Network; they were shipped overnight to the laboratory in sterile tissue culture medium and were processed as described below. Procurement of specimens from the Cooperative Human Tissue Network was also approved via our Institutional Review Board. Specimens were embedded in paraffin without decalcification.

### Sand rat intervertebral disc tissue

Animal studies were performed following approval by the Institutional Animal Care and Use Committee. *Psammomys obesus*, the sand rat, is used in our laboratory for studies of IVD degeneration. Colony housing and animal diet descriptions have been published previously [13,14].

Spines from seven animals were used in the present study of the immunolocalization of BDNF. The spines were removed immediately following animal euthanization, and the IVDs were harvested, fixed in 10% neutral buffered formalin, decalcified, and embedded in paraffin so that *en face *sections of the disc could be obtained. Sections were processed for immunohistochemical localization of BDNF as described below.

### BDNF immunohistochemistry performed on human intervertebral disc tissue and determination of the percentage of positive cells

Paraffin sections were cut at 4 μm, collected on PLUS slides (Allegiance, McGaw Park, IL, USA), dried at 60°C, deparaffinized in xylene (Allegiance) and rehydrated through graded alcohols (AAPER, Shelbyville, KY, USA) to distilled water. Antigen retrieval was performed using Dako Target Retrieval Solution, pH 6.0 (Dako, Carpenteria, CA, USA) for 20 minutes at 95°C followed by cooling for 20 minutes. The remainder of the procedure was performed using the Dako Autostainer Plus (Dako) automated stainer. Endogenous peroxidase was blocked using 3% H_2_O_2 _(Humco, Texarcana, TX, USA). Slides were incubated for 1 hour with antihuman BDNF (clone number 35928; R&D Systems, Minneapolis, MN, USA) at a 1:20 dilution. Mouse IgG (Dako) was used as a negative control. The secondary antibody was Dako LSAB2 biotinylated Link for HRP/AP (Dako) for 10 minutes followed by peroxidase-conjugated streptavidin (Dako) for 10 minutes and DAB (Dako) for 5 minutes. Slides were removed from the stainer, rinsed in water, counterstained with light green, dehydrated, cleared and mounted with resinous mounting media.

The proportion of cells positive for BDNF localization was determined for the outer annulus, the inner annulus and the nucleus. For quantitation, the average total numbers of cells scored were as follows (mean ± standard deviation): outer annulus, 387 ± 110 (n = 22); inner annulus, 390 ± 154 (n = 6), and nucleus, 331 ± 43 (n = 4).

### BDNF immunohistochemistry performed on sand rat intervertebral disc tissue

Immunohistochemistry was performed as described above with the following exceptions. The antibodies were biotinylated using the Dako ARK Kit (Dako). Sections were incubated with streptavidin peroxidase (Dako ARK Kit) for 15 minutes at room temperature and were then incubated in DAB (Dako) for 5 minutes. Rinses were performed between the application of each reagent on the AutostainerPlus using Tris-buffered saline containing Tween-20 (Dako). Slides were removed from the stainer, rinsed in water, counterstained with 0.03% light green (Polysciences, Warrington, PA, USA), dehydrated cleared and mounted with Cytoseal XYL (Richard-Allan Scientific, Kalamazoo, MI, USA).

### BDNF-receptor immunolocalization

Embedding, sectioning and blocking endogenous peroxidase using 3% H_2_O_2 _were performed as described above. Slides were then incubated for 1 hour with rabbit antibody to tyrosine kinase receptor B (BDNF receptor, batch number Rb68-010906-WS; Biosensis, Flagstaff Hill, Australia) at a 1:500 dilution. Universal negative control rabbit (Dako) was used as a negative control. The secondary antibody was Dako LSAB2 biotinylated Link for HRP/AP (Dako) for 10 minutes followed by peroxidase-conjugated streptavidin (Dako) for 10 minutes and DAB (Dako) for 5 minutes. Slides were removed from the stainer, rinsed in water, counterstained with light green, dehydrated, cleared and mounted with resinous mounting media.

### Human intervertebral disc cell culture

Annulus cells were grown in monolayer culture (seeded at 150,000 cells/well) on a Lab-Tek^® ^Chamber Slide™ (Nunc, Napierville, IL, USA) or in three dimensions in a collagen sponge as previously described [15,16].

### Gene expression studies

Human IVD tissue, annulus cells in a monolayer, and annulus cells in three-dimensional culture were assayed for gene expression using the Affymetrix microarray system (Affymetrix, Santa Clara, CA 95051, USA). Harvested cells were placed in extraction buffer from the PicoPure RNA Isolation Kit (Arcturus, Mountainview, CA, USA), and total RNA was extracted from the tissue using the PicoPure RNA Isolation Kit following the manufacturer's instructions, were reverse transcribed to double-stranded cDNA, were subjected to two rounds of transcription, and were hybridized to the DNA microarray in the Affymetrix Fluidics Station 400 (Affymetrix, Santa Clara, CA 95051, USA). Affymetrix human U133 X3P arrays were used (Affymetrix, Santa Clara, CA 95051, USA). The GeneChip^® ^Operating Software Affymetrix GeneChip Operating System (version 1.2; Affymetrix, Santa Clara, CA 95051, USA) was used for determining gene expression levels.

### Statistical analysis

The statistical analysis utilized standard methods using SAS software (version 8.2; SAS Institute, Cary, NC, USA). Methods used included unpaired *t *tests and Spearman's correlation statistics. *P *< 0.05 was considered statistically significant. Data are expressed as the mean ± standard error of the mean, or mean ± standard deviation where noted.

## Results

Immunohistochemical specimens used in the localization of BDNF and of the BDNF receptor are described in Table 1. Thirty-one IVDs from 22 subjects were evaluated; three subjects were control donors, and 19 subjects were patients undergoing disc surgery. The study design included discs within each of the Thompson grade classifications. Since the majority of the surgical specimens we obtain come from grade III and grade IV discs, there were fewer grade I and grade V discs in the present study.

**Table 1:** Demographic features of specimens studied for immunocytochemical localization of brain-derived neurotrophic factor and of its receptor

Subject number	Site	Thompson score	Age (years), gender	Other information	Herniated?
1	L4 to L5: OA, IA, nuc	1.5	45, female	Cooperative Human Tissue Network – unknown cause of death	-
	L3 to L4: OA, IA, nuc	2			-
2	L4 to L5	2	35, female	Surgical specimen	No
3	L4 to L5	2	32, female	Surgical specimen	Yes
4	L5 to S1	2	21, male	Surgical specimen	Yes
5	L4 to L5	2	44, female	Surgical specimen	Yes
6	L4 to L5	2	37, female	Surgical specimen	Yes
7	L4 to L5: OA, IA, nuc	2.5	40, male	Cooperative Human Tissue Network – cause of death, myocardial infarct	-
8	L4 to L5	3	46, female	Surgical specimen	No
9	L3 to L4	3	53, male	Surgical specimen	Yes (recurrent)
10	L4 to L5	3	53, male	Surgical specimen	No
11	L4 to L5, L5 to S1	3	29, female	Surgical specimen	No
12	L4 to L5	3	54, male	Surgical specimen	Yes
13	L2 to L3	3	58, male	Surgical specimen	Yes
14	L1 to L2: OA, IA, nuc	3	33, female	Cooperative Human Tissue Network – cause of death, pulmonary embolism	-
14	T12 to L1: OA, IA, nuc	3.5			-
14	L3 to L4: OA, IA, nuc	4			-
15	L1 to L2, L2 to L3, L3 to L4	4	59, female	Surgical specimen	No
16	L4 to L5	4	39, female	Surgical specimen	Yes (recurrent)
17	L5 to S1	4	48, female	Surgical specimen	Yes (recurrent)
18	L2 to L3, L3 to L4, L4 to L5, L5 to S1	4	56, female	Surgical specimen	No
19	L4 to L5	4	19, male	Surgical specimen	No
20	L3 to L4	4	78, male	Surgical specimen	Yes
21	L5 to S1	5	44, female	Surgical specimen	No
22	L5 to S1	5	39, male	Surgical specimen	No

IA, inner annulus; L, lumbar; nuc, nucleus; OA, outer annulus; L, lumbar.

### BDNF immunolocalization

**Figure 1:**
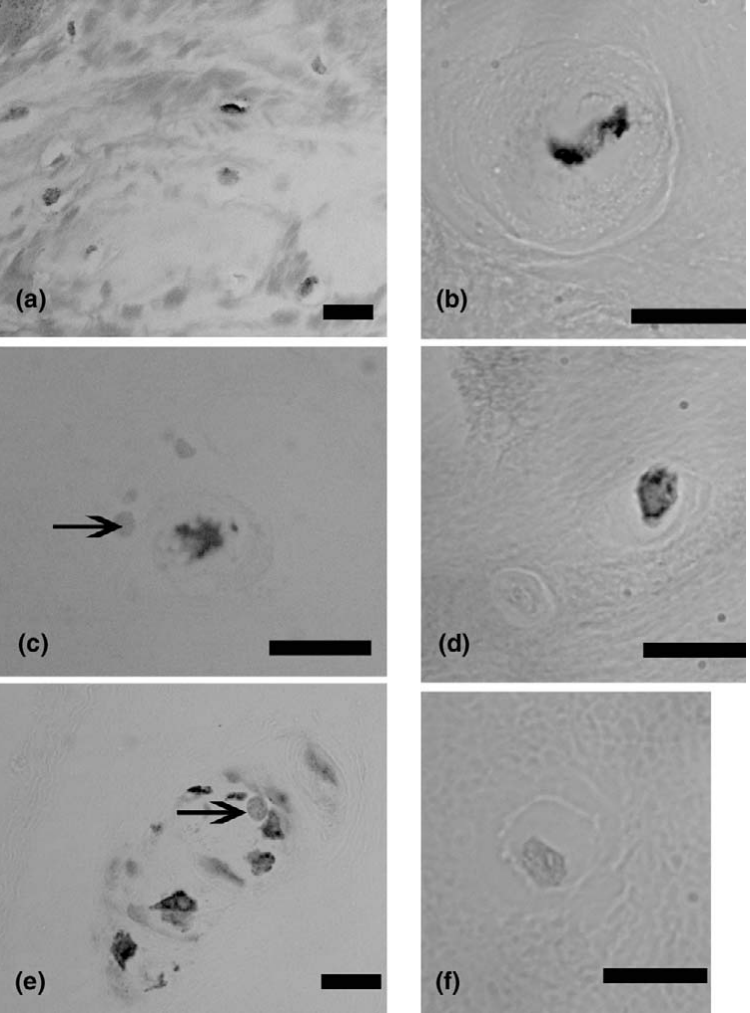
**Immunolocalization of brain-derived neurotrophic factor in human intervertebral discs**. **(a) **Outer annulus, subject number 14, L3 to L4. **(b) **Outer annulus, subject number 4, L1 to L2. **(c) **Nucleus, subject number 14, L3 to L4 (arrow marks nearby negative cell). **(d) **Outer annulus, subject number 4, L1 to L2 (note nearby cell that does not show localization). **(e) **Inner annulus, subject number 14, a cluster of cells (arrow marks one of the negative cells that lies near the positive cells). **(f) **Negative control. Bars: (a) 10 μm, (b) 0.1 μm, (c) 10 μm, (d) 10 μm, (e) 10 μm, (f) 10 μm.

**Figure 2:**
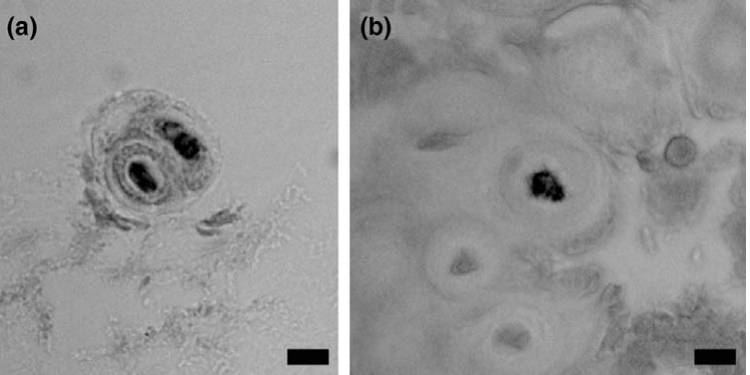
**Immunolocalization of brain-derived neurotrophic factor in annulus cells with prominent encapsulating extracellular matrix**. **(a) **Inner annulus, subject number 6, L4 to L5. **(b) **Inner annulus, subject number 12, L4 to L5. Bars = 10 μm.

Immunolocalization of BDNF was greatest in the outer annulus (Figure 1a,b). Localization of positive cells next to negative cells was also observed in the outer annulus, the inner annulus and the nucleus (Figure 1c to 1e; Figure 1f is a negative control). Positive and negative cells were present within small clusters (Figure 1e). Annulus cells encapsulated by the extracellular matrix also showed positive localization (Figure 2).

**Figure 3:**
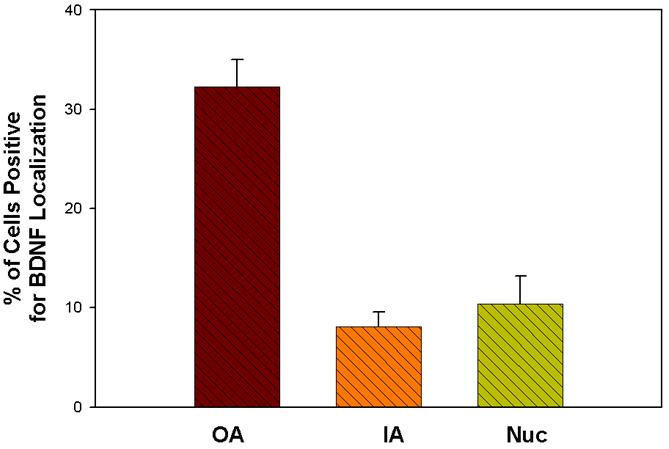
**Cells positive for brain-derived neurotrophic factor localization**. Mean percentages of cells positive for brain-derived neurotrophic factor (BDNF) localization are shown here for the outer annulus (OA, n = 22), the inner annulus (IA, n = 6), and the nucleus (nuc, n = 4). The mean percentage in the OA is significantly greater than that present in IA or in nuc (*P *≤ 0.01). Data presented as the mean ± standard error of the mean.

**Figure 4:**
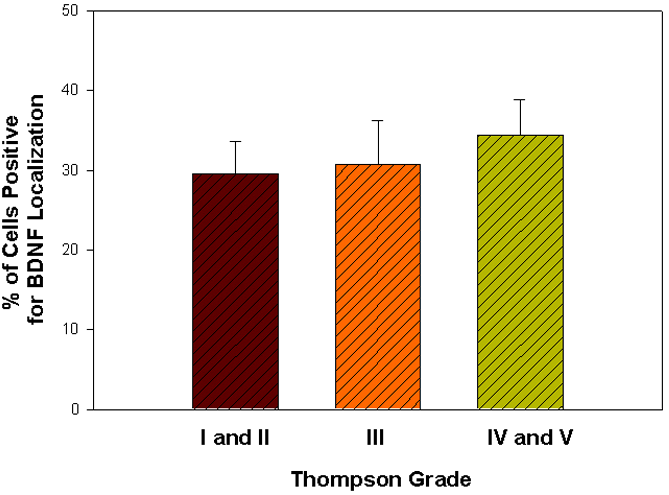
**Outer annulus cells positive for brain-derived neurotrophic factor localization by Thompson grade**. Mean percentage of outer annulus cells positive for brain-derived neurotrophic factor (BDNF) localization is shown for specimens from Thompson grades I and II pooled (n = 5), for grade III (n = 6), and for grades IV and V pooled (n = 11). Data presented as the mean ± standard error of the mean.

The proportions of BDNF-positive cells were quantitatively determined for the outer annulus, the inner annulus and the nucleus pulposus. The outer annulus contained an average of 32.3 ± 2.7% (n = 22) (mean ± standard error of the mean) cells positive for BDNF, a significantly greater level than that present in the inner annulus (8.1 ± 1.5%, n = 6) or in the nucleus (10.4 ± 2.8%, n = 4) (*P *≤ 0.01) (Figure 3). When data for the outer annulus were assessed based on the degenerative stage of the IVD (Thompson grade), no significant relationship was present (Figure 4).

**Figure 5:**
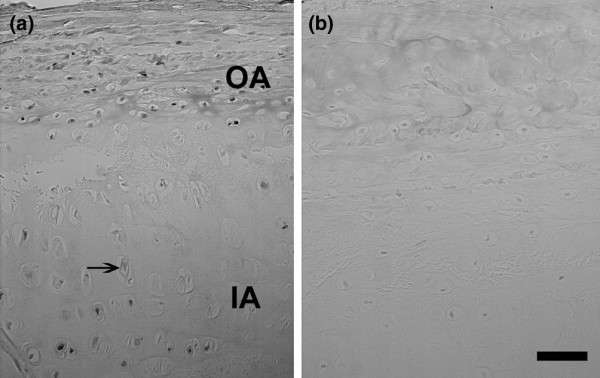
**Immunolocalization of brain-derived neurotrophic factor in sand rat intervertebral discs**. **(a) **Immunolocalization of brain-derived neurotrophic factor in a lumbar sand rat intervertebral disc. OA, outer annulus; IA, inner annulus; arrow, negative cell in the IA. **(b) **Negative control. Bars = 50 μm.

Sand rat lumbar IVDs sectioned *en face *showed a similar pattern of localization of BDNF. The greatest number of positive cells was present in the outer annulus, with fewer positive cells as one viewed the inner annulus (Figure 5).

### BDNF-receptor immunolocalization

**Figure 6:**
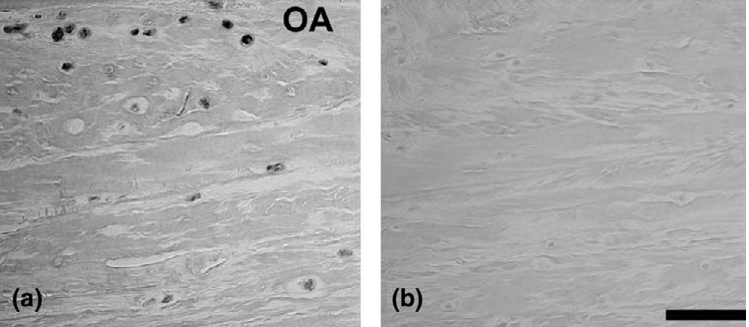
**Immunolocalization of brain-derived neurotrophic factor receptor in human intervertebral discs**. **(a) **Immunolocalization of brain-derived neurotrophic factor receptor in subject number 7, L4 to L5. OA, outer annulus. **(b) **Negative control. Bars = 50 μm.

**Figure 7:**
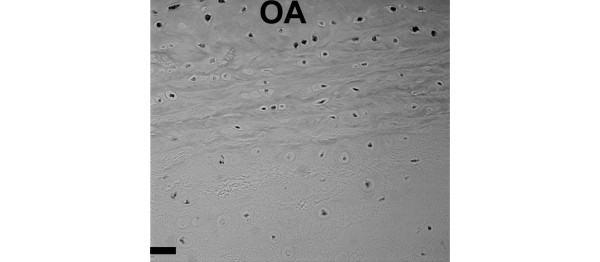
**Immunolocalization of brain-derived neurotrophic factor receptor in sand rat intervertebral discs**. Immunolocalization of brain-derived neurotrophic factor receptor in a lumbar sand rat intervertebral disc. OA, outer annulus. Bar = 25 μm.

Localization patterns were similar for the BDNF receptor to that seen for BDNF. Both positive cells and negative cells were present, with the greatest number of positive cells present in the outer annulus (Figure 6). Positive cells were occasionally present in the nucleus in human specimens. Sand rat lumbar IVDs showed a similar localization pattern, with a larger proportion of positive cells in the outer annulus compared with the inner annulus or the nucleus (Figure 7).

### Gene expression of BDNF by annulus cells

The final portion of the present study looked at gene expression of BDNF by human annulus cells cultured in monolayer and three dimensions. Annulus cells were all derived from surgical patients. This experiment utilized one grade II disc (patient aged 45 years), five grade III discs (mean patient age 38.6 years), and four grade IV discs (mean patient age 52.2 years). All cells were cultured for 10 days prior to cell harvest. Positive expression levels were observed in all specimens for BDNF (gene identifier NM_001709.1) per microarray analysis.

**Figure 8:**
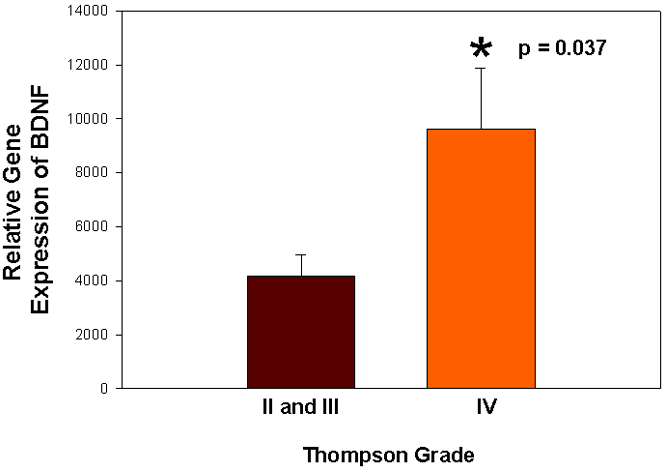
**Gene expression levels of brain-derived neurotrophic factor in cultured human annulus cells versus Thompson grades**. Graphical presentation of relative gene expression levels of brain-derived neurotrophic factor (BDNF) in cultured human annulus cells versus Thompson grades. Intervertebral discs of grades II and III were pooled (n = 6) and compared with intervertebral discs of grade IV (n = 5). Relative gene expression is significantly greater in the grade IV specimens (*P *= 0.037). Data presented as the mean ± standard error of the mean.

The BDNF gene expression levels correlated significantly and in a positive manner with the Thompson disc grade for the cell cultures (*P *= 0.011, *r *= 0.72). When expression levels in grades II and grade III discs (the healthier discs in this evaluation) were pooled and compared with mean expression levels in grade IV discs, significantly higher expression was identified in the more degenerate IVDs (*P *= 0.037; Figure 8).

## Discussion

The present study presents novel immunocytochemical data demonstrating the presence of BDNF and of BDNF receptor in the human outer annulus, inner annulus and nucleus pulposus. Quantitative assessment of the proportion of cells positive for BDNF localization showed significantly greater localization in the outer annulus compared with the inner annulus or the nucleus pulposus (*P *≤ 0.01). Quantitative analysis was not performed for the BDNF receptor, but qualitatively its localization appeared to follow a similar pattern.

BDNF and the BDNF receptor were also found to be present in sand rat lumbar IVDs, and the immunolocalization pattern was similar to that seen in human IVDs but with more labeled cells present in the outer annulus. Inner annulus cell and nucleus cells in the human IVD were found to occasionally show positive immunolocalization, but at a significantly lower proportion. Although there was minimal background present in the animal tissues, it must be remembered that this portion of our study utilized antihuman antibodies, no biochemical analysis of the cross-reactivity of the sand rat substrates was carried out, and a danger of some false-positive reactivity with proteins in the sand rat IVD remains a concern that at present cannot be discounted.

Neurotrophins are beginning to be viewed as important agents in the IVD. Aoki and colleagues have suggested that NGF-dependent neurons may be the nerve population responsible for discogenic pain based on findings from studies of disc degeneration in rats [17,18]. A study of human discs from subjects with low back pain performed by Freemont and colleagues identified a positive relationship between ingrowth of nonmyelinated, nociceptive nerves into the IVD and the production of nerve growth factor by nearby ingrowing blood vessels [19].

Studies have recently shown production of several neurotrophins by IVD cells, but the mechanisms that control their expression, and the effects these factors have on disc cells, are poorly understood. Gigante and colleagues recently reported the presence of NGF mRNA and the high-affinity tyrosine kinase A receptor and the low-affinity p75 receptor in the rounded cells in the disc annulus and nucleus pulposus [5]. The p75 receptor is of special interest because it also has the ability to bind to BDNF [20].

The work presented here adds to, and expands, our understanding of the distribution of BDNF and its receptor in the human IVD. In addition to NGF, BDNF has recognized neuroprotective effects [21]. There is also evidence that BDNF can be considered a neurotransmitter and may have a role in nociceptive transmission [22]. Work by Thompson and colleagues suggests that BDNF may be released from primary sensory nociceptors with activity such as that involved with persistent pain states and inflammatory conditions [9]. It is well recognized that differential regulation of neurotrophin expression occurs in inflammatory states, such as in the airway, where NGF, BDNF and neurotrophin 3 have been shown to be elevated during inflammation [23]. Increased proinflammatory cytokines are now well recognized during IVD degeneration [24-27], providing support for the hypothesis that BDNF *in vivo *may be produced in response to inflammatory cytokines during IVD degeneration. Clearly the relationship of BDNF to inflammatory cytokines in the IVD merits future research attention.

The recent studies of by Kermani and colleagues regarding the ability of BDNF to promote revascularization via endothelial cells and hematopoietic progenitors are currently unexplored in the IVD [11]. These investigators suggest that the signaling pathway involving BDNF and its receptor may function in modulation of angiogenesis in specific organs in the adult. Since the adult disc is avascular, the potential of BDNF to influence angiogenesis in the mature disc is intriguing.

Finally, the initial observations by Boyd and colleagues that showed the upregulation of BDNF by application of increased osmolarity remains intriguing [1], and suggests the potential importance of the unexplored relationship between mechanoregulation and BDNF in the human IVD.

## Conclusion

The data presented here document the presence of BDNF and its receptor in the human IVD at the translational level, and also show the expression of BDNF and its receptor by cultured human annulus cells. Quantitative assessment of the proportion of cells positive for BDNF localization showed that the outer annulus contained a significantly larger proportion of positive cells compared with the inner annulus or the nucleus (*P *≤ 0.01). Our findings point to the need for further studies to define the role of BDNF in the human IVD and to investigate regulatory events, especially those related to mechanoregulation, which regulate the expression of BDNF and its receptor in the human IVD.

## Abbreviations

BDNF = brain-derived neurotrophic factor; IVD = intervertebral disc; IL = interleukin; NGF = nerve growth factor; TNF = tumor necrosis factor.

## Competing interests

The authors declare that they have no competing interests.

## Authors' contributions

HEG conceived of the study and participated in study design with ENH, analyzed the data, and prepared and revised the manuscript. JAI, GH and NZ performed laboratory work and analysis. HJN performed statistical analyses and assisted with manuscript preparation. All authors approved the final manuscript.
